# Pancreatic fat content by magnetic resonance imaging in subjects with prediabetes, diabetes, and controls from a general population without cardiovascular disease

**DOI:** 10.1371/journal.pone.0177154

**Published:** 2017-05-17

**Authors:** Sophia D. Heber, Holger Hetterich, Roberto Lorbeer, Christian Bayerl, Jürgen Machann, Sigrid Auweter, Corinna Storz, Christopher L. Schlett, Konstantin Nikolaou, Maximilian Reiser, Annette Peters, Fabian Bamberg

**Affiliations:** 1 Department of Diagnostic and Interventional Radiology, University of Tuebingen, Tuebingen, Germany; 2 Institute of Clinical Radiology, Ludwig-Maximilian-University Hospital, Munich, Germany; 3 Section on Experimental Radiology, Department of Diagnostic and Interventional Radiology, University Hospital Tuebingen, Tuebingen, Germany; 4 Institute for Diabetes Research and Metabolic Diseases (IDM) of the Helmholtz Center Munich at the University of Tuebingen, Germany; 5 German Center for Diabetes Research (DZD), Tuebingen, Germany; 6 Department of Radiology, Diagnostic and Interventional Radiology, University of Heidelberg, Heidelberg, Germany; 7 German Center for Cardiovascular Disease Research (DZHK e.V.), Munich, Germany; 8 Institute for Cardiovascular Prevention, Ludwig-Maximilian-University-Hospital, Munich, Germany; 9 Institute of Epidemiology II, Helmholtz Zentrum München, German Research Center for Environmental Health, Neuherberg, Germany; University of Oslo, NORWAY

## Abstract

**Background/Objective:**

Despite the relevance of pancreatic fat content in the development of metabolic diseases, its association with impaired glucose metabolism, diabetes, and other adipose tissue compartments remains unclear. Thus, we determined differences in pancreatic fat content by magnetic resonance imaging (MRI) between subjects with prediabetes, diabetes, and normal controls in a cohort from the general population.

**Methods:**

Subjects without history of cardiovascular disease with established diabetes or prediabetes as well as normal controls were included and underwent whole-body MRI on a 3T scanner. Pancreatic fat content was quantified by measuring the proton-density fat fraction (PDFF_panc_) using a 3D multi-echo GRE sequence (increment: 1.23 ms, 6 echoes) by placing ROIs in the pancreatic head, body, and tail by independent readers. In addition, hepatic fat content as well as abdominal subcutaneous and visceral adipose tissue (SAT and VAT) were measured by multi-echo GRE and 3D 2-point volume-interpolated DIXON MRI, respectively. Univariate and multivariate analyses were employed to determine associations.

**Results:**

A total of 385 subjects were included in the analysis (median age: 57 years, 58.2% males), of them 53 were classified as subjects with diabetes, 95 as prediabetes, and 237 as controls (13.8%, 24.7%, and 61.6%; respectively). The median PDFF_panc_ was 5.2% [IQR 3.3–9.4], and significantly higher in subjects with prediabetes and diabetes as compared to controls (PDFF_panc_: 6.2% [IQR: 3.5–12] vs. 8.6% [IQR: 4.3–17.5] vs. 4.9% [3.1–7.4], p<0.001, respectively). After adjusting for age, gender and BMI the association was attenuated (all p>0.12). While in univariate analysis BMI, PDFF_hepatic_, SAT and VAT were associated with PDFF_panc_ (all p<0.05), only VAT predicted PDFF_panc_ independently (β: 0.02, 95%-confidence interval: 0.01–0.04, p<0.001).

**Conclusion:**

While pancreatic fat content differs significantly between subjects with prediabetes, diabetes and controls, this association may be confounded by age, gender, and the amount of VAT in this cross-sectional study.

## Introduction

While it is well established that diabetes mellitus is associated with increased cardiovascular morbidity and mortality [1, 2], higher risk of hospitalization [3], and a substantial healthcare burden [4], there is a large group of subjects not yet classifying as diabetic but already presenting with impaired glucose metabolism [5]. This group of subjects with prediabetes exhibits not only increased rates of progression to diabetes mellitus but also carries a significant risk of cardiovascular disease and may therefore may represent a valuable prevention target [6]. While obesity plays a central role in the disease process, there is increasing evidence that local fat depots, such as abdominal visceral adipose tissue (VAT) rather than general adiposity can be linked with impaired glucose metabolism [7, 8]. However, the specific role of the different fat depots in the development of prediabetes and diabetes is still not fully understood.

This is particularly relevant for the accumulation of ectopic fat in the pancreas, also known as fatty pancreas [9]. Pancreatic fat content may play a role in several local pathological processes such as pancreatic cancer or subtypes of pancreatitis [10, 11]. In addition, available data suggest that decreased pancreatic volume and increased pancreatic fat content are more frequently observed in subjects suffering from impaired glucose metabolism [12, 13] and pancreatic fat content was reported to correlate with insulin secretion in subjects at increased risk for metabolic diseases [14]. Larger studies covering greater numbers of participants report rather inconsistent results on a direct association of pancreatic fat content and impaired glucose metabolism [15, 16]. One explanation of these heterogeneous findings may be the different imaging modalities used for the assessment of pancreatic fat content, including ultrasound, computed tomography (CT), and magnetic resonance imaging (MRI) [15–20]. Given its non-ionizing nature and high soft tissue contrast, MRI may be particularly suited to gain insights into the role of pancreatic fat content [17].

Thus, the objective of this study was to determine differences in pancreatic fat content as measured by MRI between subjects with prediabetes, diabetes, and normal controls in a cohort from the general population. In addition, findings were compared with other fat depots, including hepatic fat content, subcutaneous, and visceral adipose tissue. Our hypothesis was, there are differences in pancreatic fat content between subjects with prediabetes, diabetes, and healthy controls.

## Methods

### Study design

The “Cooperative Health Research in the Region of Augsburg” (KORA) study was designed as a nested, prospective case-control study in the southern part of Germany [21]. Among subjects enrolled in the KORA-FF4 cohort, eligible subjects with prediabetes, diabetes, and controls underwent whole-body MRI. The study was approved by the local institutional review board of the Ludwig-Maximilian-University Munich and informed written consent was obtained from all participants. The detailed study protocol as well as the inclusion and exclusion criteria are described elsewhere [22]. Briefly, subjects without contraindications to MRI and without history of prior cardiovascular disease (such as prior percutaneous coronary intervention, myocardial infarction or bypass graft, peripheral artery disease, or stroke), who were classified as either diabetic, prediabetic, or normal controls were eligible. The imaging protocol included MR sequences for characterization of the cardiovascular and metabolic system. All subjects also underwent a comprehensive assessment for the presence of cardiovascular risk factors at the study center.

### Covariates

All covariates were obtained from actual measurements during the study visit. For classifying subjects into the three subgroups, an oral glucose tolerance test was performed for all subjects not yet being diagnosed with diabetes. Diabetes was defined as fasting glucose ≥7.0mmol/l (126 mg/dl) and/or 2–h serum glucose ≥11.1mmol/l (200 mg/dl) according to WHO recommendations [23]. Similarly, prediabetes was defined as either impaired glucose tolerance (IGT) with a normal fasting glucose and a 2-hour glucose between >7.8 and <11.1 mmol/l (140 mg/dl and 200 mg/dl) and/or an impaired fasting glucose (IFG) with a fasting glucose 6.1–6.9 mmol/l (110 mg/dl– 125 mg/dl) and a 2-hour glucose <7.8 mmol/l (140 mg/dl). Normal controls were classified by the absence of either diabetes or prediabetes (2-hour serum glucose under 140 mg/dl and fasting glucose under 110 mg/dl) [23].

Relevant cardiovascular risk factors were collected as part of the KORA study protocol [21]. In brief, BMI was calculated as weight (kg) divided by height squared (m^2^). Hypertension was defined as a systolic blood pressure ≥140 mmHg and diastolic blood pressure ≥ 90 mmHg or the intake of antihypertensive medication. Alcohol consumption was classified according to the anamnesis of drinking no alcohol at all (0 g/day), moderate alcohol consumption (0.1–39.9 g/day for men and 0.1–19.9 g/day for women) and heavy alcohol consumption (≥40 g/day for men and ≥ 20 g/day for women) [24]. Smoking status was defined as never-, ex- and current smoker. Medication being antihypertensive by most recent guidelines was defined as ‘antihypertensive medication’ and lipid-lowering medication was defined as the routinely intake of statins, fibrates or other lipid lowering agents.

### MR imaging protocol

Whole-body MRI examinations were performed on a 3 Tesla Magnetom Skyra MRI (Siemens Healthcare, Erlangen, Germany). All subjects underwent an identical imaging protocol on the same MR scanner. The complete imaging protocol including technical details is provided elsewhere [22]. As part of the imaging protocol, a 3D multi-echo Dixon sequence [25] of the upper abdomen was employed for the assessment of pancreatic and hepatic fat content which included the following parameters: time-to-repetition (TR) 8.90 ms, time-to-echo (TE) 1 1.23 ms (opposed-phase), TE2 2.46 ms (in-phase), TE3 3.69 ms (opposed-phase), TE4 4.92 ms (in-phase), TE5 6.15 ms (opposed-phase), TE6 7.38 ms (in-phase), flip angle 4°, partition thickness 4mm, field-of-view (FOV) read 420mm, and FOV phase 78.1%. Acquisitions were obtained during a breath-hold of approximately 15 seconds. These measurements account for confounders such as T2* decay, T1 bias, noise bias and fat composition [26], resulting in the relative proton density fat fraction (PDFF). An automated calculation and output of a stack with quantitative coding of PDFF in degrees of gray values was performed and archived to the PACS-system.

For quantification of SAT, and VAT parameters, a two point Dixon gradient-echo (GRE) sequence was employed with the following parameters: TR 4.06 ms, TE 1.26, 2.49 ms, flip angle 9°, partition thickness 1.7 mm, isotropic in-plane resolution 1.7 mm. These measurements also account for hemosiderin deposition using R2* within a single breath-hold.

### MR image analysis

Image analysis was performed on a dedicated off-line workstation by readers unaware of the diabetes status or any other information pertaining to the risk status of the subjects.

#### Pancreatic fat content

**Fig 1:**
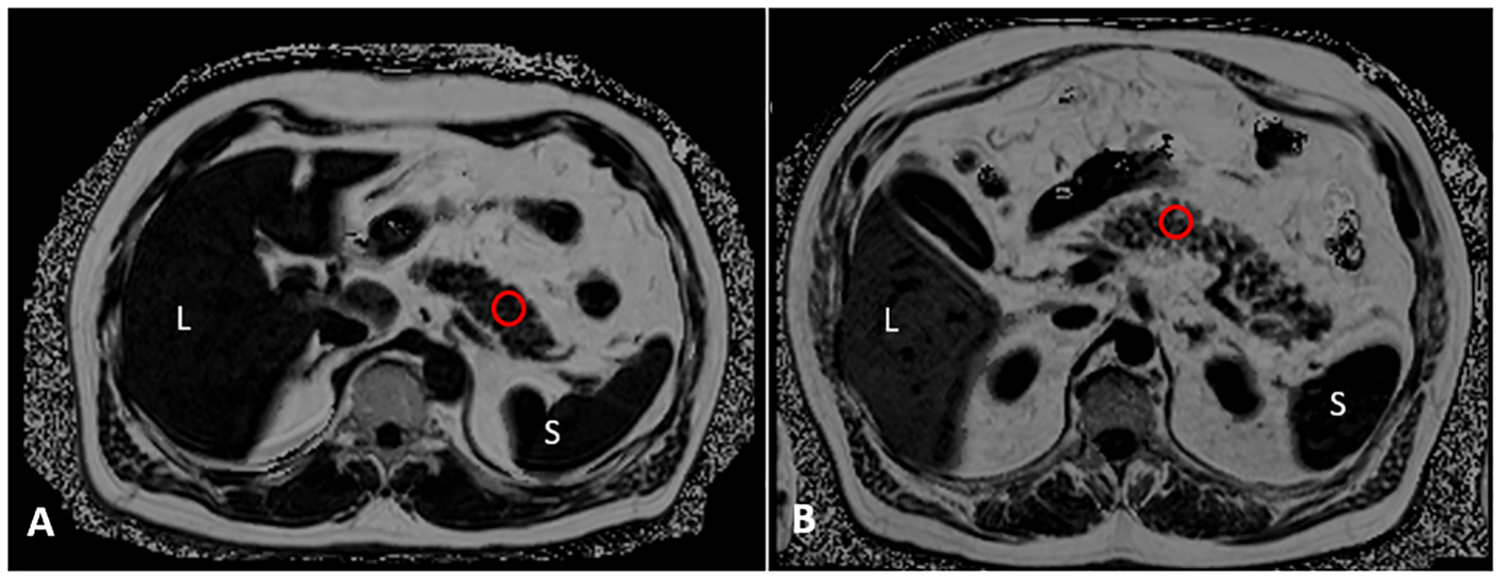
Assessment of pancreatic fat content in subjects with lower and higher pancreatic fat content undergoing 3T magnetic resonance imaging (multi-echo GRE Dixon sequence) from the general population. Pancreatic fat content was measured as proton-density fat fraction (PDFF_panc_) in a region of interest (red circle). L = liver; S = spleen.

For quantitative assessment of pancreatic fat content (measured as proton-density fat fraction [PDFF_panc_]), circular regions of interest (ROI) covering an area of approximately 100 mm^2^ were drawn into the pancreatic head (caput), the pancreatic body (corpus) and the pancreatic tail (cauda) in different MRI-slices (Fig 1) using a dedicated off-line workstation (Syngo Via, Siemens Healthcare, Erlangen, Germany) [19, 27]. Images with severe image artifacts (e.g. phase swaps) were excluded from the analysis. The data were recorded in a database. Inter-reader and intra-reader variability was assessed in a subset of 40 subjects. Intra- and interobserver variability was low (ICC: 0.95 95%-CI 0.90 to 0.97 and ICC: 0.80, 95%-CI: 0.66 to 0.89; respectively). The reliability of PDFF measurements has previously been validated [15].

#### Hepatic fat content

Based on the acquired multi-echo Dixon images of the upper abdomen, hepatic fat content measured as PDFF_hepatic_ was derived from a single axial slice at the level of the portal vein. As such, a ROI was drawn into the liver parenchyma, carefully avoiding inclusion of visible extra- and intrahepatic vessels, and absolute PDFF_hepatic_ was calculated. Again, images with significant artifacts were excluded from the analysis [28].

#### SAT and VAT

Abdominal adipose tissue compartments were estimated from a single axial slice at the umbilical level, as it has been shown that measurements in this slice are representative for the total amount of VAT location [29]. This slice was reconstructed from the 3D VIBE-Dixon images which were assessed in coronal direction. Axial slices were reconstructed with a slice thickness of 5mm. SAT and VAT were segmented applying an automated procedure based on fuzzy-clustering [30].

### Statistical analysis

Descriptive characteristics of the study group are presented as median (25^th^ and 75^th^ percentile [interquartile range, IQR]) or absolute numbers (percent values). Differences between healthy controls, participants with prediabetes and diabetes were assessed by Kruskal-Wallis equality-of-populations rank test (quantitative data) or χ^2-^test (qualitative data). Differences of outcome parameters of pancreatic fat content among diabetic status groups were additionally assessed by test for trend and displayed by box-and-whisker plots. Agreements of pancreatic fat content parameters of the caput, corpus and cauda within the groups were investigated by Friedman's analysis of variance test. Correlations of pancreatic fat content with VAT, SAT, PDFF_hepatic_ and BMI were demonstrated by scatter plots and Spearman’s rho correlation coefficients were provided.

Effects of prediabetes and diabetes status as well as other cardiovascular risk factors for skewed distributed pancreatic fat content were estimated median with 95% confidence intervals (CI) from quantile regression in unadjusted models. Associations between diabetic status and pancreatic fat content were further adjusted for age, sex and BMI using multivariable quantile regression.

A two-sided p-value of <0.05 was considered to indicate statistical significance. Statistical analyses were performed using Stata 14.1 (Stata Corporation, College Station, TX, U.S.A.).

## Results

Of 400 enrolled subjects, 385 subjects were included in the present analysis with complete image acqusition and sufficient image quality (96.25%); they were predominantly middle aged men (median age: 57 [IQR: 48–64] years; 58.2% males). Among them, 53 were classified as diabetic, 95 as prediabetic, and 237 as controls (13.8%, 24.7%, and 61.6%; respectively). Detailed demographics are provided in Table 1. Subjects with diabetes were generally older, more likely male, and had a higher BMI, whereas control subjects were youngest, more likely female, and with lowest BMI (all p<0.05). Subjects with prediabetes ranged in between controls and subjects with diabetes with respect to cardiometabolic risk profiles.

**Table 1:** Demographics of the KORA study population. Data are given as number (percentage) or median (25^th^ and 75^th^ percentile).

Characteristics	All subjects	Controls	Prediabetes	Diabetes	P
N	385	237	95	53	
Age (years)	57 (48; 64)	53 (47; 62)	59 (51; 66)	63 (58; 69)	<0.001
Sex (men)	224 (58.2%)	122 (51.5%)	62 (65.3%)	40 (75.5%)	0.002
BMI (kg/m^2^)	27.4 (24.7; 30.9)	26.2 (23.7; 28.9)	29.7 (27.3; 33.8)	30.4 (27; 33)	<0.001
Hypertension	133 (34.6%)	51 (21.5%)	44 (46.3%)	38 (71.7%)	<0.001
Systolic blood pressure (mmHg)	121 (109; 131)	116 (107; 126)	124 (117; 134)	133 (118; 144)	<0.001
Diastolic blood pressure (mmHg)	75 (69; 81)	74 (68; 80)	78 (72; 85)	79 (72; 84)	<0.001
Triglyceride levels (mg/dl)	108 (77; 155)	94 (69; 126)	145 (99; 186)	177 (113; 269)	<0.001
Total cholesterol (mg/dl)	217 (191; 240)	215 (190; 242)	225 (201; 244)	200 (183; 232)	0.02
HDL (mg/dl)	60 (48; 72)	62 (51; 77)	58 (47; 68)	48 (41; 62)	<0.001
LDL (mg/dl)	138 (117; 160)	136 (116; 162)	145 (124; 162)	130 (109; 150)	0.03
Lipid lowering medication	40 (10.4%)	15 (6.3%)	7 (7.4%)	18 (34%)	<0.001
Anti-hypertensive medication	99 (25.7%)	41 (17.3%)	31 (32.6%)	27 (50.9%)	<0.001
PDFF_hepatic_ (%)	4.7 (2.7; 12)	3.4 (2.1; 6)	11.6 (4.8; 17.9)	15 (6.7; 24.1)	<0.001
VAT (cm^2^)	144 (81; 206)	99.2 (57; 152)	182 (143; 241)	216.8 (193; 289)	<0.001
SAT (cm^2^)	256.5 (201; 342)	239.9 (184; 309)	293 (236; 393)	297.8 (226; 373)	<0.001

Prediabetic and diabetic subjects had a significantly higher amount of PDFF_hepatic_, VAT, and SAT compared to healthy controls and were more often under lipid-lowering and anti-hypertensive medication. There was no difference of lifestyle factors, such as alcohol consumption and smoking between the groups.

### Pancreatic fat content by MRI

**Fig 2:**
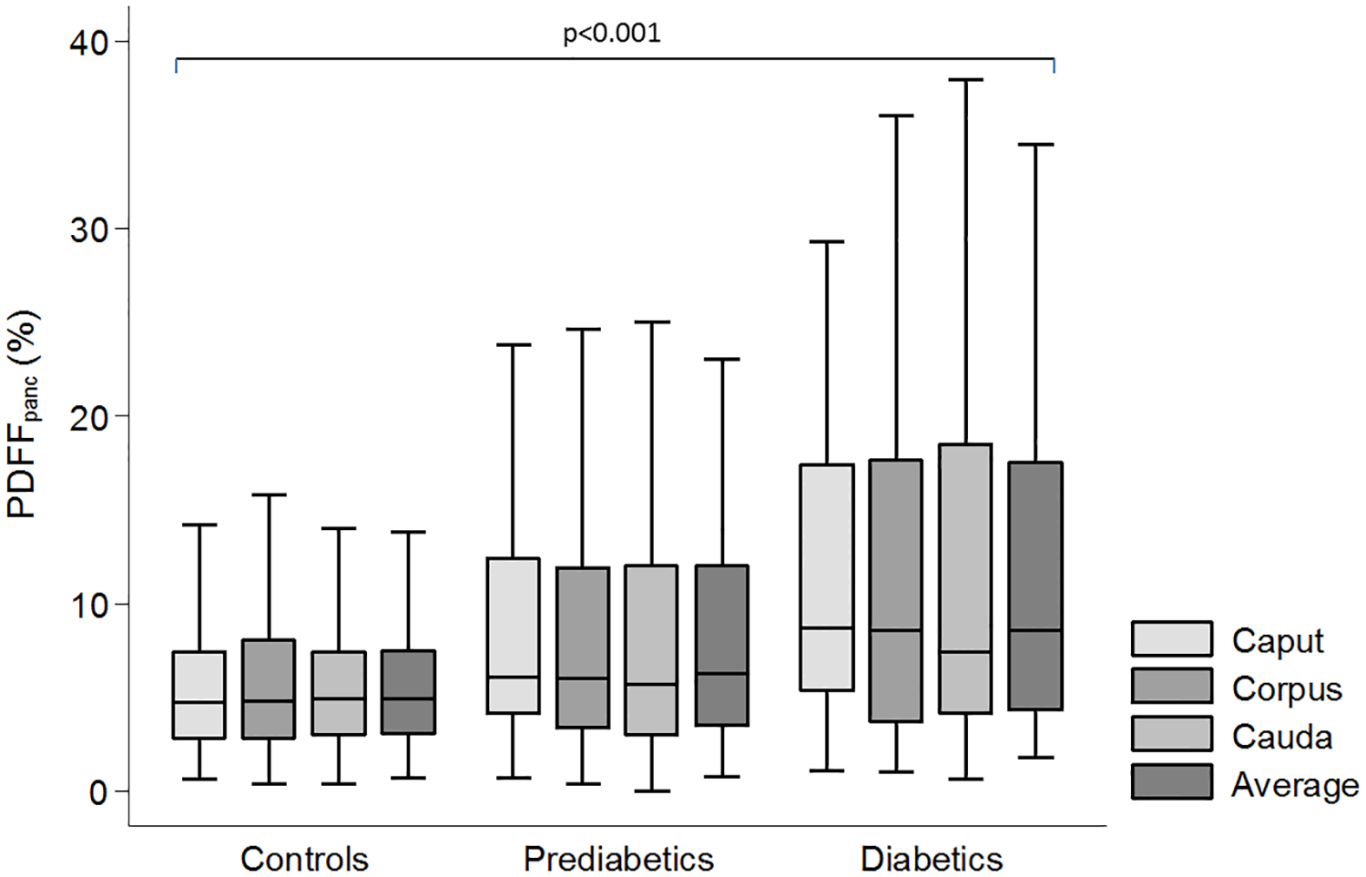
Differences of pancreatic fat content between controls, subjects with prediabetes and diabetes displayed by box-and-whisker.

The median of average PDFF_panc_ in all subjects was 5.2% [IQR: 3.3–9.4] and there was no significant difference with respect to mesurements obtained in the caput, corpus or cauda (Fig 2; all p>0.05). PDFF_panc_ was significantly higher in the prediabetic (PDFF_panc_ 6.2% [IQR: 3.5–12]) and highest in the diabetic (PDFF_panc_ 8.6% [IQR: 4.3–17.5]) subjects in comparison with healthy controls (p-value for trend: <0.001). These differences for prediabetes and diabetes were also observed in groupwise comparison (p = 0.045 and p<0.001 for prediabetes and diabetes as compared to controls, respectively). After adjusting for age, gender and BMI, the observed differences for PDFF_panc_ between subjects with prediabetes, diabetes, and controls were attenuated (β: -0.43, 95%-CI: -1.84–0.98 and β: 1.4, 95%-CI -0.38–3.18 for prediabetes and diabetes, respectively).

### Predictors of pancreatic fat content

**Fig 3:**
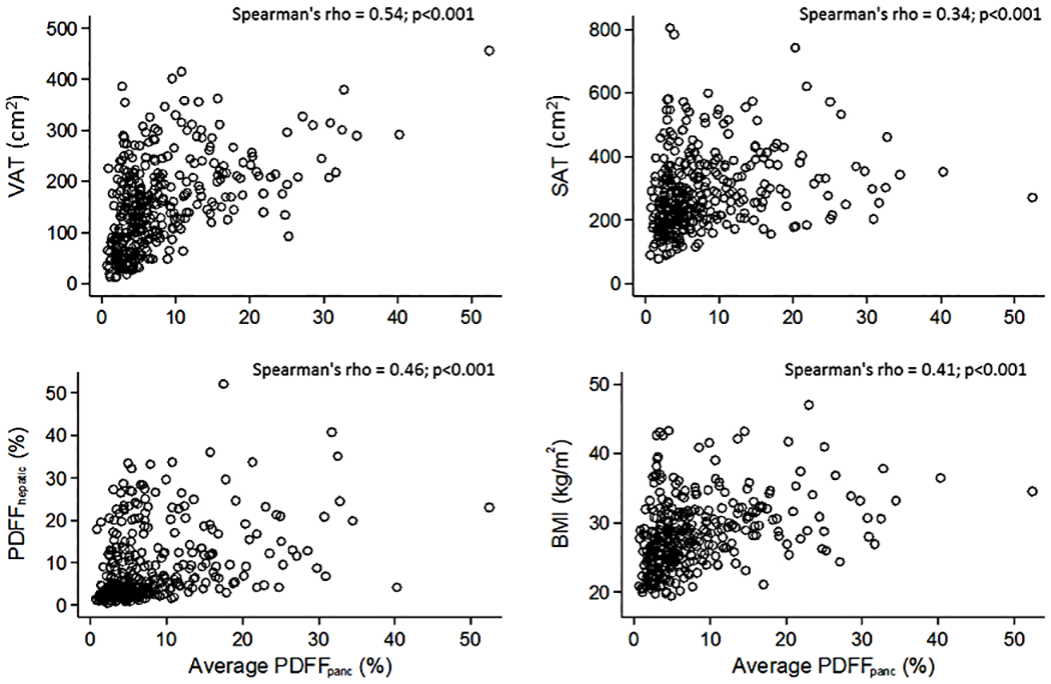
Scatter plots for correlations of pancreatic fat content with VAT, SAT, PDFF_hepatic_ and BMI.

In univariate analysis, the majority of established cardiometabolic risk factors, including age, male gender, hypertension and triglyceride levels were significantly and positively associated with PDFF_panc_ (Table 2). Moreover, BMI, SAT, VAT, and PDFF_hepatic_ correlated significantly with PDFF_panc_ (Fig 3), while total cholesterol, LDL-concentration, and lifestyle factors such as current smoking, moderate and heavy alcohol consumption were not significantly associated with PDFF_panc_. In contrast, higher levels of HDL, the intake of lipid-lowering medication as well as anti-hypertensive medication was associated with lower amounts of PDFF_panc_.

**Table 2:** Univariate analysis of associations between demographic and cardiometabolic risk factors and pancreatic fat content. β-coefficients derived from median regression, CI, confidence interval. PDFF: proton-density fat fraction.

Predictor	Estimate (Beta)	95%-CI	P-value
Age (years)	0.13	0.07–0.18	<0.001
Male gender	1.20	0.14–2.26	0.03
BMI	0.39	0.28–0.49	<0.001
Diabetes Status			
• Control	Reference		
• Prediabetics	1.30	0.03–2.57	0.045
• Diabetics	3.63	2.05–5.22	<0.001
Hypertension	2.03	0.76–3.30	0.002
Systolic blood pressure (mmHg)	0.06	0.03–0.09	<0.001
Diastolic blood pressure (mmHg)	0.07	0.02–0.12	0.003
Triglyceride levels (mg/dl)	0.02	0.01–0.02	<0.001
Total cholesterol (mg/dl)	0.01	-0.01–0.02	0.39
HDL (mg/dl)	-0.04	-0.06–-0.01	0.006
LDL (mg/dl)	0.01	0; 0.03	0.08
PDFF_hepatic_ (%)	0.2	0.14–0.27	<0.001
VAT (cm^2^)	0.03	0.02–0.04	<0.001
SAT (cm^2^)	0.01	0.01–0.02	<0.001
Lipid lowering medication	-2.1	-3.69–-0.51	0.01
Anti-hypertensive medication	-1.77	-3.11–-0.43	0.01
Smoking status			
• Never-Smoker	Reference		
• Ex-Smoker	1.3	0.17–2.43	0.02
• Current-Smoker	-0.07	-1.47–1.34	0.93
Alcohol consume (g/day)			
• No	Reference		
• Moderate	-0.23	-1.42–0.95	0.70
• Heavy	0.1	-1.3–1.5	0.89

After adjusting for SAT, VAT and PDFF_hepatic_, as well as other potential confounders, only VAT remained a significant predictor of PDFF_panc_ (β: 0.02, 95%-CI 0.01–0.04), whereas particularly the associations for PDFF_hepatic_, SAT and BMI became non-significant (Table 3).

**Table 3:** Multivariate associations between demographics, cardiometabolic risk factors and pancreatic fat content.

Predictor	Average PDFF_panc_(%)	
	β (95% CI)	P-value
Age (years)	0.03 (-0.04; 0.10)	0.419
Male gender	-0.05 (-1.49; 1.38)	0.942
BMI	0.09 (-0.18; 0.36)	0.513
Diabetes Status		
• Control	Reference	
• Prediabetics	-0.80 (-2.29; 0.69)	0.290
• Diabetics	-0.27 (-2.36; 1.83)	0.803
Hypertension	0.69 (-0.67; 2.04)	0.318
Triglyceride levels (mg/dl)	0.00 (-0.01; 0.01)	0.938
HDL (mg/dl)	0.01 (-0.03; 0.05)	0.682
LDL (mg/dl)	0.01 (-0.01; 0.02)	0.571
PDFF_hepatic_ (%)	-0.01 (-0.10; 0.08)	0.882
VAT (cm^2^)	0.02 (0.01; 0.04)	<0.001
SAT (cm^2^)	0.00 (-0.01; 0.01)	0.812
Lipid lowering medication	0.15 (-1.89; 2.19)	0.885
Smoking status		
• Never-Smoker	Reference	
• Ex-Smoker	0.5 (-0.75; 1.75)	0.432
• Current-Smoker	-0.25 (-1.83; 1.32)	0.750

## Discussion

In this cohort from the general population, our results demonstrate that there are differences in pancreatic fat content between subjects with prediabetes, diabetes, and normal controls, with a continuous increase in PDFF_panc_ from controls, to prediabetes, to subjects with established diabetes. However, our results also indicate that these associations are not independent of other established risk factors, predominantly age, gender and BMI. Moreover, when taking into account other ectopic fat compartments, the effect on pancreatic fat content may be predominantly confounded by visceral adipose tissue. Thus, our results emphasize the role of visceral adipose tissue in the development of a hyperglycemic metabolism.

It is well established that ectopic fat compartments play a central role in the development of metabolic disease states [31]. Major interest has been raised to pancreatic fat content, given its focal accumulation in the insulin-producing organ and presumably effect on endocrine function [14]. However, while early research was hampered by limited assessment of the pancreas by reliable techniques, recent research has been fostered by the implementation of advanced imaging modalities [12, 19, 27]. Previous research on pancreatic fat content has suggested that it is increased in hyperglycemic metabolic states [13] but also that there may be less differences than initially anticipated [15]. In a sample from the general population including subjects with prediabetes, diabetes, and normal controls, we now provide more detailed knowledge on that specific topic.

Our results show that a median of average PDFF_panc_ of 5.2% can be assumed across all groups. This finding is in line with *Kuhn et al*., who found a mean unadjusted PDFF of 4.4% in subjects from the SHIP-study in Northern Germany [15]. Also, similar values (average pancreas PDFF 5.7%) were reported by *Idilman et al*. in 41 subjects with biopsy-proven non-alcoholic fatty liver disease (NAFLD) [17]. While we confirm these prior observations, our results also demonstrate that there is a continuous increase in PDFF_panc_ ranging from healthy controls to subjects with prediabetes to diabetes. Similar observations have also been made by *Dong et al*. [12] who describe an increase in pancreatic fat content in subjects with impaired glucose metabolism as assessed by MRI in 83 subjects.

However, after adjustment for age and gender, these differences between controls and subjects with prediabetes were attenuated. While it is well known that pancreatic fat content is higher in elderly people and in men [9, 15, 32], differences were maintained for subjects with diabetes, potentially indicating the more advanced stage of disease with detectable morphological changes [13]. When additionally adjusting for BMI, the observed differences for pancreatic fat content in diabetic subjects were also attenuated. As such, our findings are in line with *Kuhn et al*., who did not find a relation of pancreatic fat content to impaired glucose metabolism [15]. Indirect confirmation stems from a recent longitudinal study showing that pancreatic fat content was not associated with the development of diabetes in a 5-year cohort study [33]. Moreover, a lack of association between pancreatic fat content and impaired beta-cell function has been reported earlier [34, 35].

In the present study, we now provide more detailed insights into the independent associations of pancreatic fat content and other ectopic fat compartments as measured by MRI. Specifically, we found VAT, SAT, and PDFF_hepatic_ were higher in subjects with prediabetes and diabetes. Our observed differences of ectopic fat compartments between subjects with prediabetes, diabetes and controls are in line with previous research. For instance, *Neeland et al*. found that in 732 obese subjects from the Dallas Heart study excess visceral fat was associated with incident prediabetes and type 2 diabetes [7].

Notably, we found that among the three examined adipose compartments, VAT remained the only independent predictor of pancreatic fat content. The association between VAT and pancreatic fat content has been described earlier [14, 32, 36]. For instance, *Rossi et al*. found that VAT by MRI was the strongest predictor for pancreatic fat content in a study comprising 12 lean and 38 obese subjects [32]. We now confirm this finding in a significantly larger cohort. VAT has recently been identified as a major risk factor for metabolic disease states and cardiovascular disease [37, 38] and our study contributes to the acknowledgement of the central role of VAT in the development of metabolic disease states.

With VAT being the only independent predictor of PDFF_panc_ in multivariate analysis, it is striking that the existing associations of other potential factors, including PDFF_hepatic_ and BMI were attenuated in multivariate analysis. Again, this finding may highlight the central role of VAT in the pathogenesis of metabolic disease states [38]. Clearly, our results confirm the inferior role of BMI as compared with a more detailed imaging-based assessment of body composition (including VAT) as a crude estimate of body composition [39].

As the predominant effect of VAT in hypermetabolic states is substantial, at least from a clinical point of view, it may be subject to discussion whether clinical implementation of assessment of VAT may be beneficial in a selected set of subjects at increased risk for metabolic diseases in order to develop beneficial health care programs [40]. First studies presenting automated assessment of MRI-derived fat depots resulted in promising findings [30]. However, further evidence and dedicated cost-effectiveness-analyses are certainly needed.

Interestingly, we did not detect any independent association between lipid-lowering medication and PDFF_panc_ beyond VAT whereas lipid lowering medication had a protective effect in univariate analysis. In a 10 year follow up study it has been shown that lipid lowering medication was not associated with a risk to develop type 2 diabetes mellitus [41]. Similar observations concerning the non-existence of an independent association were made by us with respect to smoking status, potentially indicating the inert role of pancreatic fat content to this external factor. However, smoking is a risk factor for pancreatic cancer [42]. A beneficial effect of subgroups of antihypertensive medication, such as valsartan (angiotensin type 1 receptor blocker), in the metabolic syndrome has been suggested [43], nevertheless we mainly focused on the more obvious interactors of pancreatic fat content such as BMI, VAT, SAT and PDFF_hepatic_.

Our study has some limitations. First, the analysis cohort represents a sample from a healthy population in Germany, thus generalizability to other, particularly non-European populations may be limited. Our groups of individuals were not matched adequately in order to compensate for differences beyond the presence of pancreatic fat content (i.e. age and gender). However, differences were accounted for using multivariate analysis adjusting for all differences detected in univariate analysis. Despite that we applied multivariate adjustment in our analysis, it needs to be highlighted that this approach may induce collinearity (i.e. between pancreatic fat and VAT) and a true increase in pancreatic fat may be falsely attenuated by VAT. Thus, further confirmatory, more homogeneously matched group comparisons are warranted. Notably, not all subjects were firstly diagnosed as prediabetes and diabetes and the majority of subjects were under medication according to current guidelines. However, as such the study cohort represents a very representative sample from a western European population and we have adjusted for these differences using multivariate analysis. In addition, we did not define a cut-off value for the definition of pancreatic steatosis but employed the average PDFF_panc_ values. While this may be in contrast to other approaches, it provides more pathophysiological insights into the disease process. While we applied an established method of measuring pancreatic fat content by PDFF[15, 17, 19], it may be that visceral adipose tissue may have contaminated these measurements. However, while our approach is in line with previous research and measurements carefully avoided the inclusion of adjacent visceral fat, further more detailed assessment of pancreatic tissue compartments is warranted. We also assessed pancreatic fat content manually, which may limit the opportunity to apply the approach to larger cohorts and samples. However, more advanced post-processing techniques are currently being developed, which may overcome the need for manual segmentation in the near future [30].

**In conclusion**, our results indicate that PDFF_panc_ is significantly higher in subjects with prediabetes and diabetes as compared to healthy controls. However, this association may be confounded by age, gender, and the amount of VAT in this cross-sectional study.
